# Molecular epidemiology, antibiotic resistance profile and frequency of integron 1 and 2 in adherent-invasive *Escherichia coli* isolates of colorectal cancer patients

**DOI:** 10.3389/fmicb.2024.1366719

**Published:** 2024-06-13

**Authors:** Aida Heidari, Mohammad Hassan Emami, Fatemeh Maghool, Samane Mohammadzadeh, Parisa Kadkhodaei Elyaderani, Tahereh Safari, Alireza Fahim, Razie Kamali Dolatabadi

**Affiliations:** ^1^Poursina Hakim Digestive Diseases Research Center, Isfahan University of Medical Sciences, Isfahan, Iran; ^2^Physiology Department, School of Medicine, Zahedan University of Medical Sciences, Zahedan, Iran; ^3^Department of Medicine, Najafabad Branch, Islamic Azad University, Najafabad, Iran; ^4^Clinical Research Development Center, Najafabad Branch, Islamic Azad University, Najafabad, Iran

**Keywords:** adherent-invasive *Escherichia coli*, colorectal cancer, antimicrobial resistance, integron, MLST

## Abstract

This study explores the prevalence of adherent-invasive *Escherichia coli* (AIEC) in colorectal cancer (CRC) patients and investigates the potential of effective intracellular antibiotics as a therapeutic strategy for CRC patients with AIEC infections. Considering the pivotal role of integrons in bacterial antibiotic resistance, the frequency of class 1 and 2 integrons in AIEC isolated from CRC patients, in one of the referenced 3 gastroenterology clinics in Isfahan, Iran was examined. AIEC strains were isolated from the colorectal biopsies and their antimicrobial sensitivity was assessed using the disc diffusion method. Polymerase chain reaction (PCR) was employed to detect *intl1* and *intl2*. The multilocus sequence typing (MLST) method was utilized to type 10 selected isolates. Of the 150 samples, 24 were identified as AIEC, with the highest number isolated from CRC2 (33.4%) and CRC1 (29.16%), and the least from the FH group (8.3%) and control group (12.5%). *int1* in 79.2% and *int2* in 45.8% of AIEC strains were found and 41.6% of strains had both integrons. AIEC isolates with int1 exhibited the highest sensitivity to trimethoprim-sulfamethoxazole (57.9%), while those with int2 showed the highest sensitivity to ciprofloxacin (63.6%). A significant association between resistance to rifampin and integron 2 presence in AIEC isolates was observed. Furthermore, a significant correlation between integron 1 presence, invasion, survival, and replication within macrophages in AIEC strains was identified. MLST analysis revealed ST131 from CC131 with integron 1 as the most common sequence type (ST). The emergence of such strains in CRC populations poses a serious public health threat. The distribution pattern of STs varied among studied groups, with pandemic STs highlighting the importance of examining and treating patients infected with these isolates. Comprehensive prospective clinical investigations are warranted to assess the prognostic value of detecting this pathovar in CRC and to evaluate therapeutic techniques targeting drug-resistant AIECs, such as phage therapy, bacteriocins, and anti-adhesion compounds, for CRC prevention and treatment.

## 1 Introduction

Adherent-invasive *Escherichia coli* (AIEC) pathotype was first identified in 1999 in a patient with Crohn’s disease, a form of chronic inflammatory bowel disease (IBD). Since then, this pathotype has been reported in other diseases such as colorectal cancer (CRC). According to the World Health Organization, about 25% of malignancies are caused by infections. Given that CRC ranks as the third most common cancer and the second leading cause of death worldwide in 2020, it is important to investigate the infectious factors involved in the development of this malignancy (Rawla and Barsouk, 2019). Genetic factors elucidate about 20% of the total CRC incidence, while environmental factors encompass the remaining 80% of CRC cases. The composition of microorganisms in the gut ecosystem is among the environmental factors that may contribute to CRC etiology. Several studies have suggested composition of the gut microbiota could impact human health, and gastrointestinal microbial dysbiosis has been observed in CRC patients (Kamali Dolatabadi et al., 2022).

Martin et al. have demonstrated a relationship between AIEC and the pathogenesis of CRC. Given the established role of AIEC and other pathogens in the development of CRC as demonstrated in several studies, antibiotics targeting intracellular bacteria such as quinolones, rifampin, and trimethoprim-sulfamethoxazole, can be considered a logical therapeutic approach in CRC patients (López-Siles et al., 2022). Surgery and chemotherapy have long been the conventional therapies for CRC. However, with the notable advancements seen in targeted therapy, including the efficacy of specialized drugs and agents, targeted treatment strategies are rapidly emerging (Xie et al., 2020). Some studies have shown that targeted antibiotic therapy with clarithromycin, rifampin, and ciprofloxacin may be effective in inducing and maintaining remission and preventing the risk of recurrence following surgical intervention in CRC patients (Fedorak and Ismond, 2016). Therefore, antibiotic therapy is also suggested for managing CRC patients alongside other therapeutic interventions (Rawla and Barsouk, 2019). So far, there has been a limited study investigating the antibiotic resistance profiles of AIEC pathovars (Mousavifar et al., 2018; Martinez-Medina et al., 2020; Cho et al., 2022). Dugan et al. have investigated antimicrobial resistance in AIEC and non-AIEC strains, revealing the presence of beta-lactam and integron 1 resistance genes among Crohn’s disease patients (Dogan et al., 2012). Therefore, considering the possible relationship between CRC infection and exacerbation in the presence of AIEC, understanding antimicrobial resistance and the underlying molecular mechanisms in these strains becomes crucial. In recent years, due to the arbitrary and uncontrolled use of antibiotics, antibiotic resistance is increasing. Several studies have documented the prevalence of integrons and heightened antibiotic resistance among *E. coli* isolates (Halaji et al., 2020). Integrons are mobile genetic elements that carry antibiotic resistance genes. Integrons consist of conserved 3′ and 5′ regions and variable regions between these two regions. The 5′ conserved regions include the common promoter (Pc), the integrase gene (IntI), and the recombination site (attI), and the protected parts 3′ have sul1 and qacEΔ1 genes, which contain genes for resistance to sulfonamides and specific detergents (Shin et al., 2015). Integrons are divided into different classes from integrons 1 to 4 based on the differences in the integrase amino acid sequence. Integrons 1, 2, and 3 are carried on mobile genetic regions and integron 4 is carried on chromosomal regions (Bhat et al., 2023). Various antibiotic resistance gene coding sequences, including resistance genes for more than 130 different antibiotics, have been identified on gene cassette regions. Class 1 integron has been the most widespread and commonly reported among clinical bacteria (Shetty et al., 2023). Its prevalence is usually reported between 22 and 59% in clinical Gram-negative bacteria (Deng et al., 2015).

The amino acid sequences of the intI2 gene exhibit less than 50% similarity compared to intI1., and replacement of the internal terminal codon with glutamic acid may result in an inactive and short polypeptide that is non-functional and incapable of catalyzing the recombination reaction (Ramatla et al., 2023). The origin of this stop codon remains unidentified, yet it exerts a regulatory function and creates another type of integrase. Due to the simultaneous carrying of class 1 and 2 antigens, the small number of different gene cassette arrays, and the low diversity of cassette genes have formed this hypothesis. Although the ability to pick up and integrate site-specific attI2 gene cassettes, intI2 cannot attC sites of gene cassettes from the class1 recognize the integron for further integration. In this way, class 2 integrons share gene cassettes such as dfrA1, sat1, and aadA1 with class 1 integrons (Deng et al., 2015). In the classic structure of class 2 integrons resistance to trimethoprim, streptothricin, and streptomycin/spectinomycin, resulting in a dihydrofolate reductase (dfrA1), streptothricin acetyltransferase (sat1) and aminoglycoside adenyl transferase (aadA1) gene cassettes.

Researchers have categorized four types of integrons based on the integrase gene in Gram-negative bacteria, and class 1 and 2 integrons exhibiting higher prevalence among clinically resistant isolates (Joddha et al., 2023). Considering the important role of integrons in bacterial resistance to antibiotics and increasing drug resistance, this study aimed to investigate the frequency of class 1 and 2 integrons in AIEC isolated from CRC patients within one of the referenced 3 gastroenterology clinics in Isfahan, Iran.

## 2 Methods

### 2.1 Patients and tissue samples

From our previous study, a collection of 74 intracellular *E. coli* strains was isolated from specimens of colorectal biopsies of 150 participants including patients with CRC [CRC1, carcinoma *in situ* (Tis); CRC2, tumor stage (T1-T2 or T3); CRChis, patients who had a history of CRC that were referred for a 6-month follow-up; FH, individuals who have a family history of CRC, and the healthy control group]. Adhesion and Invasion Assay and Intramacrophage Cell Survival Test were carried out as previously described, and 24 strains were identified as AIEC pathotypes (Kamali Dolatabadi et al., 2022). The study was approved by the Ethics Committee of Isfahan University of Medical Sciences (approval no. IR.ARI.MUI.REC.1401.124). All patients provided written informed consent to participate. Individuals who did not take any antibiotics for a month before the colonoscopy were included in the study.

### 2.2 Cell culture

Human colorectal adenocarcinoma (Caco-2) cell lines with identification number: NCBIC10094 and murine macrophage-like cell line J774A.1 with identification number: NCBIC483 were purchased from the cell bank of the National Genetic Reserve Center of Iran.

The Caco-2 cell line was used to evaluate the adhesion and invasion ability of *E. coli* strains inside cells and the J774A.1 cell line was applied to assess the survival and replication ability of macrophages (Kamali Dolatabadi et al., 2022). Cell lines were cultured on Dulbecco’s modified Eagle’s medium (DMEM) DMEM-F12 supplemented with 10% FBS and 2 mM amino acid L-glutamine along with 1% penicillin–streptomycin antibiotic and were transferred to an incubator at °C 37 and 5% CO2 and 95% humidity (Fazeli and Hasan Emami, 2021).

### 2.3 Adhesion test

To perform the adhesion test, the Caco2 cell line was inoculated with a dilution of 5 * 10^5^ cells in each well on a 24-well plate after 48 h of incubation, then the cells were infected with 0.5 mL of *E. coli* isolates in the logarithmic phase of growth ([10^6^bacteria/ml, a multiplicity of infection (MOI) of 10]), and were incubated for 3 h at 37°C under 5% CO2 pressure and 95% humidity. Then, the monolayer cells were washed three times with PBS buffer and then lysed with 20 μL of 1% Triton X-100 with a concentration of 0.1%.

The resulting suspension was diluted in PBS buffer and cultured on an LB agar medium to calculate the number of adherent bacteria. Cultivation on the plate was done quickly within 30 min to prevent the lysis of bacteria by Triton X-100. The adhesion index (I-ADH) was calculated as the ratio of the bacterial count per cell to the total number of initially inoculated bacteria (Dalmasso et al., 2019).

### 2.4 Invasion test

For conducting this assay, the Caco2 cell lines were inoculated with a dilution of 5 * 10^5^ cells in each well on a 24-well plate. Then the cells were infected with *E. coli* isolates that had adhesion ability based on the previous step with MOI = 10. Infected cells were incubated for 3 h at 37°C. After incubating, the cells were washed again with PBS buffer (pH = 7.2), and the fresh culture medium containing 100 μg/mL of gentamicin was incubated for another 1 h (Gentamicin protection assay) to remove extracellular bacteria. Then, with the method mentioned in the previous step (adhesion test), the cells were lysed using 1% Triton X-100 and the number of invasive bacterial cells was calculated. The invasion index (I-INV) was determined as the percentage of harvested bacteria relative to the total initial inoculated bacteria. An isolate was considered invasive if I-INV% ≥ 0.1% (Dalmasso et al., 2019). *Shigella sonnei* (ATCC 9290) was used as a positive control and *E. coli* K12 was used as a negative control for the adhesion and invasion test (Martin et al., 2004).

### 2.5 Survival and replication test inside the macrophage

This test was performed using the J774A-1 macrophage cell line. The *E. coli* isolates assessed for their adhesion and invasion capabilities were further evaluated for their capacity to persist and proliferate within macrophages. J774A-1 macrophage cell line was inoculated with a concentration of 1*10^5^ cells in each well in a 24-well plate and incubated for 20 h. Each well-contained DMEM medium was supplemented with 10% FBS and 2 mM amino acid L-glutamine along with 1% penicillin–streptomycin antibiotic. Then the cells were infected with *E. coli* isolates (MOI = 10). To promote the penetration of bacteria into macrophages, the samples were incubated for 3 h at 37°C with 0.5% CO2. Then, to remove non-phagocytosed bacteria, the fresh culture medium containing 100 μg/mL gentamicin was incubated for another hour (gentamicin protection assay) and the intracellular bacterial cells were determined with a method similar to the invasive test, 1 h and 24 h after infection (Camprubí-Font et al., 2019). *Salmonella enterica* (ATCC 9270) was used as a positive control and *E. coli* K12 was used as a negative control for the survival and replication test inside the macrophage (Martin et al., 2004). I-REPL calculated using this equation:

$I-\mathrm{REPL}\;\left(\%\right)=\left(CFU\;\mathrm{ml}-1\;\mathrm{at}\;24\;\mathrm{hr}/CFU\;\mathrm{ml}-1\;\mathrm{at}\;1\;\mathrm{hr}\right)*100$

Strains with INV% > 0.1% and I-REPL% = 100% were considered AIEC strains (Dalmasso et al., 2019).

### 2.6 Determining antibiotic sensitivity

In this study, the antimicrobial sensitivity of the isolates against antibiotics known for their efficacy against intracellular microorganisms such as rifampicin, trimethoprim, sulfamethoxazole, ciprofloxacin and so on was determined based on the disc diffusion method using Muller-Hinton (MH) agar medium according to the instructions of the Clinical and Laboratory Standards Institute (CLSI; Rai et al., 2023).

### 2.7 Molecular analysis

Bacterial DNA was extracted from fresh bacterial colonies according to the previously described method (Chen and Kuo, 1993). Polymerase chain reaction (PCR) was performed to detect the presence of intl1 and intl2 using the specific primers (Table 1). PCR amplification conditions were as follows: initial denaturation at 94°C for 5 min followed by 35 cycles of denaturation at 94°C for 45 s, primer annealing at 62°C for intI1 and 58°C for intI2, elongation at 72°C for 45 s, and final elongation at 72°C for 7 min. PCR products were separated by electrophoresis on a 1% agarose gel with 1x buffer (EDTA/Acetate/Tris) at 65 volts and for 2 h. Green-violet dye was used for staining and products were observed under ultraviolet light.

**Table 1:** Primers used for PCR analysis.

Primer sequences (5′_3′)	Target sequences	Amplicon size	References
**Virulence genes**			
F-GGTCAAGGATCTGGATTTCG	U49101 (int1)	483 bp	Fayyazi et al. (2020)
R-ACATGCGTGTAAATCATCGTC			
F-CACGGATATGCGACAAAAAGGT	L10818 (int2)	789 bp	Fayyazi et al. (2020)
R-GTAGCAAACGAGTGACGAAATG			

### 2.8 Phylogenetic analysis and multilocus sequence typing

Multilocus sequence typing (MLST) was performed to assess the 10 selected AIEC isolates according to the Achtman scheme using amplification of the seven housekeeping genes including adk, fumC, gyrB, icd, mdh, purA, and recA available at MLST website.^1^ STs were assigned to clonal complex (CCs), singlelocus variants (SLV), double-locus variants (DLV), and singletons using the goeBURST algorithm using PHYLOViZ 2.0 software.

### 2.9 Statistical analysis

The statistical analysis was performed using WHONET software version 5.6 and SPSS version 24. The Chi-square and Fisher’s exact tests were used to determine any statistical relationship between variables. A *p*-value <0.05 was considered significant.

## 3 Results

### 3.1 Patient characteristics

In the present study, out of a total of 150 samples examined, 79 (52.66%) were male, and 71 (47.33%) were female. According to the information presented in Table 2. The mean age of the CRC1 group was (55.03) years, which was lower than the mean age of the CRC2 group (63.17) years. The most common indications for patients to be referred for colonoscopy according to the study groups are given in Supplementary Table S1. 61 (40.7%) of patients were referred with screening indication, 59 (39.3%) with diagnosis, and 30 (20%) with surveillance indication. As shown in Table 2 the most common indication for both CRC1 and CRC2 groups was the diagnosis, whereas in the control and FH groups, screening emerged as the predominant indication, and CRChis group, surveillance was the most common indication for colonoscopy. Table 2 shows that in CRC groups, 35% of tumors occurred in the proximal colon and 65% in the distal colon.

**Table 2:** Frequency of tumor categories by colonic segments.

	Proximal					Distal		
Groups	Ileum	Hepatic flexure	Cecum	Ascending	Transverse	Descending	Sigmoid	Rectum
CRC1 *n*(%)	1 (1.7)	2 (3.3)	0	1 (1.7)	5 (8.3)	3 (5)	6 (10)	12 (20)
CRC2 *n*(%)	0	2 (3.3)	5 (8.3)	4 (6.7)	1 (1.7)	0	5 (8.3)	13 (21.)
Total *n*(%)	1 (1.7)	4 (6.7)	5 (8.3)	5 (8.3)	6 (10)	3 (5)	11 (18.3)	25 (41.7)
	21 (35)					39 (65)		

CRC1, colorectal cancer in situ (Tis); CRC2, CRC with tumor stage T1, T2, or T3.

After processing, 74 Intracellular *E. coli* isolates were isolated from 150 samples. The isolates were from the following groups: CRC1 (24.3%), CRC2 (27%), CRChis: (18.9%), FH: (14.9%), and control: (14.9%).

24 strains out of 74 isolates of intracellular *E. coli* were identified as AIEC pathotypes based on the methods described earlier (Kamali Dolatabadi et al., 2022). The highest number of AIEC isolated from the CRC2 patient group (33.4%) was followed by the CRC1 patient group (29.16%), and the least isolated from the FH group (8.3%) and subsequently separated from the control group (12.5%; Table 3).

**Table 3:** The prevalence of AIEC isolates by studied groups.

		CRC1	CRC2	CRChis	FH	Control	*p*
Intracellular *E. coli*	AIEC = 24 *n*(%)	(16/29)7	(4/33)8	(7/16)4	(3/8)2	(5/12)9	0.7
	No AIEC = 50 *n*(%)	(22)11	(24)12	(20)10	(18)9	(16)8	

CRC1, colorectal cancer in situ (Tis); CRC2, CRC with tumor stage T1, T2, or T3; CRChis, with CRC history; FH, with a family history of CRC. Data were analyzed using the chi-square test.

### 3.2 Antibiotic resistance profile in AIEC and non-AIEC isolates

The details of the antibiotic resistance profile of the strains are given in Table 4. The highest resistance to intracellular antibiotics was seen in rifampin with a dose higher than 2 μg, and then to the antibiotic trimethoprim. In both CRC groups, the highest antibiotic resistance was seen in rifampin, followed by trimethoprim and trimethoprim-sulfamethoxazole. Regarding AIEC isolates, the highest resistance to rifampin with a dose higher than 2 μg was reported by 87.5%, followed by trimethoprim by 50%. Among all isolates, the highest sensitivity was exhibited against tetracycline 46 (62.16%) and trimethoprim-sulfamethoxazole (41 isolates, 55.4%), respectively. AIEC isolates were most sensitive to trimethoprim-sulfamethoxazole (15 isolates, 65.2%), followed by ciprofloxacin and tetracycline (11 isolates, 47.82%).

**Table 4:** Antibiotic susceptibility pattern of intracellular *Escherichia coli*.

		Number of antimicrobial resistance of intracellular *E. coli* isolates (%)									
Isolates	Group (*n*)	SXT	*p*	Trimethoprim	*p*	Ciprofloxacin	*p*	Tetracycline	*p*	Rifampin > 2	*p*
All isolates	CRC1 18	12 (66.7)	0.003	14 (77.8)	0.061	8 (44.4)	0.59	8 (44.4)	0.44	16 (88.9)	0.39
	CRC2 20	6 (30)		8 (40)		8 (40)		6 (30)		17 (85%)	
	CRChis 14	9 (64.3)		9 (64.3)		5 (35.7)		7 (50)		12 (85.7)	
	FH 11	0		2 (18.2)		2 (18.2)		2 (18.2)		10 (90.9)	
	Control 11	3 (27.3)		5 (51.4)		2 (18.2)		5 (45.5)		7 (63.6)	
	Total 74	30 (40.5)		38 (51.4)		25 (33.8)		28 (37.8)		62 (83.8)	
AIEC isolates	CRC 17	4 (57.1)	0.62	5 (71.4)	0.09	4 (57.1)	0.04	5 (71.4)	0.83	7 (100)	0.042
	CRC2 8	3 (37.5)		4 (50)		2 (25)		4 (50)		7 (87.5)	
	CRChis 4	1 (25)		2 (50)		2 (50)		2 (50)		4 (100)	
	FH 2	0		0		0		1 (50)		2 (100)	
	Control 3	1 (33.3)		1 (33.3)		0		1 (33.3)		1 (33.3)	
	Total 24	9 (37.5)		12 (50)		8 (33.3)		13 (54.2)		21 (87.5)	

AIEC, adherent-invasive *Escherichia coli*; CRC1, colorectal cancer in situ (Tis); CRC2, CRC with tumor stage T1, T2, or T3; CRChis, with CRC history; FH, with a family history of CRC.

According to Supplementary Table S2, no significant relationship was observed between the level of adherent, the percentage of invasion, the percentage of survival and replication in macrophages of AIEC isolates, as well as the percentage of antibiotic resistance observed in the isolates.

### 3.3 Integrons 1 and 2 in AIEC and non-AIEC isolates

The pattern of antibiotic sensitivity of AIEC and non-AIEC isolates with and without integrons is shown in Tables 5, 6. The antibiotic sensitivity pattern in AIEC isolates with integron 1 and integron 2 revealed that the highest antibiotic resistance to Rifampin was 89.5 and 72.7%, respectively. In AIEC isolates with integron 1, the highest sensitivity was observed to the trimethoprim-sulfamethoxazole (57.9%) and in the isolates with integron 2, the highest sensitivity was to ciprofloxacin (63.6%; Table 5). In non-AIEC isolates with integron 1, the highest sensitivity was revealed to trimethoprim-sulfamethoxazole (69%), and in non-AIEC isolates with integron 2, the most sensitivity was against tetracycline (56.7%; Table 6). Furthermore, in AIEC isolates, a significant relationship between resistance to rifampin and the presence of integron 2 was observed, while in non-AIEC isolates, a marked relationship was found between the presence of integron 2 and resistance to Trimethoprim. As shown in Table 5, there was a significant relationship between the presence of integron 1 and the invasion percentage (*p* = 0.03) and the percentage of survival and replication within the macrophages (*p* = 0.004) in AIEC strains.

**Table 5:** Antibiotic susceptibility pattern of integron-positive and integron-negative in AIEC-isolates.

Isolates				Integron-1			Integron-2		
				Positive	Negative	*p*	Positive	Negative	*p*
				*n* = 19	*n* = 5		*n* = 11	*n* = 13	
AIEC = 24	Characteristics	ADH^a^ (Mean ± SD)		1.64 ± 0.69	1.96 ± 0.54	0.34	1.7 ± 0.84	1.67 ± 0.51	0.77
		INV^b^ (Mean ± SD)		0.30 ± 0.18	0.51 ± 0.12	0.03*	0.30 ± 0.17	0.38 ± 0.21	0.32
		REPL^c^ (Mean ± SD)		240.72 ± 136.38	515.43 ± 269.13	0.004*	265.36 ± 181.24	325.52 ± 218.55	0.47
	Antibiotic	Trimethoprim-sulfamethoxazole	S	11 (57.9)	4 (80)	0.43	6 (54.5)	9 (69.2)	0.30
			R	8 (42.1)	1 (11.1)		5 (45.5%)	4 (30.8%)	
		Trimethoprim	S	7 (36.8)	4 (80)	0.26	4 (36.4)	7 (53.8)	0.27
			I	1 (5.3)	0		0	1 (7.7)	
			R	11 (57.9)	1 (91.7)		7 (63.6)	5 (38.5)	
		Ciprofloxacin	S	9 (47.4)	3 (60)	0.84	7 (63.6)	5 (38.5)	0.48
			I	3 (15.8)	1 (20)		1 (9.1)	3 (23.1)	
			R	7 (36.8)	1 (20)		3 (27.3)	5 (38.5)	
		Tetracycline	S	9 (47.4)	2 (40)	0.69	6 (54.5)	5 (38.5)	0.53
			R	10 (52.6)	3 (60)		5 (45.5)	8 (61.5)	
		Rifampin	>1	2 (10.5)	1 (33.3)	0.6	3 (27.3)	0	0.05
			>2	17 (89.5)	4 (80)		8 (72.7)	13 (100)	

Characteristics data were analyzed using one-way ANOVA and Antibiotic data were analyzed using the chi-square test, ^*^p < 0.05. S, Susceptible; I, Intermediate; R, Resistant; AIEC, adherent-invasive *Escherichia coli*. ^a^Grade of adhesion. ^b^Percentage of inoculum surviving after 1 h of gentamicin treatment (number of intracellular bacteria/initial inoculum × 100). ^c^Number of intracellular bacteria at 24 h post-infection/number of bacteria at 1 h post-infection × 100.

**Table 6:** Antibiotic susceptibility pattern of integron-positive and integron-negative in non-AIEC isolate.

Isolates	Antibiotic		Integron-1 positive	Integron-1 negative	*p*	Integron-2 positive	Integron-2 negative	*p*
			*n* = 29	*n* = 21		*n* = 30	*n* = 20	
			*n*(%)	*n*(%)		*n*(%)	*n*(%)	
Non-AIEC = 50	Trimethoprim-sulfamethoxazole	S	11 (37.9)	14 (66.7)	0.13	11 (36.7)	14 (70)	0.06
		I	3 (10.3)	1 (4.8)		3 (10)	1 (5)	
		R	15 (51.7)	6 (28.6)		16 (53.3)	5 (25)	
	Trimethoprim	S	10 (34.5)	11 (52.4)	0.44	11 (36.7)	10 (50)	0.03^*^
		I	2 (6.9)	1 (4.8)		0	3 (15)	
		R	17 (58.6)	9 (42.9)		19 (63.3%)	7 (35)	
	Ciprofloxacin	S	11 (37.9)	10 (47.6)	0.74	11 (36.7)	10 (50)	0.51
		I	7 (24.1)	5 (23.8)		7 (23.3)	5 (25)	
		R	11 (37.9)	6 (28.6)		12 (40)	5 (25)	
	Tetracycline	S	20 (69)	123 (61.9)	0.23	17 (56.7)	16 (80)	0.16
		I	0	2 (9.5)		1 (5)	1 (5)	
		R	9 (31)	6 (28.6)		12 (40)	3 (15)	
	Rifampcin	>1	4 (13.8)	5 (23.8)	0.36	6 (20)	3 (15)	0.65
		>2	25 (86.2)	16 (76.2)		24 (80)	17 (85)	

Data were analyzed using the chi-square test, ^*^p < 0.05. S, Susceptible; I, Intermediate; R, Resistant; AIEC, adherent-invasive *Escherichia coli*.

PCR results revealed the presence of integron 1 in AIEC (79.2%) and non-AIEC (59.2%) isolates, integron 2 in AIEC (45.8%) and non-AIEC (61.2%) isolates, and the simultaneous presence of integron 1 and 2 were found in AIEC (41.6%) and non-AIEC (38%) isolates (Supplementary Table S3).

### 3.4 Molecular typing of AIEC clinical isolates

AIEC strains with different phenotypic and genotypic characteristics were selected for typing from all the studied groups. After molecular typing of the selected AIEC isolates, 8 ST and 5 clonal complexes were identified among the 10 selected isolates (Table 7).

**Table 7:** A complete view of the phenotypic and genotypic characteristics of AIEC isolates.

Isolates	Group	Phylogroups	Antimicrobial resistance profiles	Integrons		I-ADH^a^ (mean)	INV^b^ (mean)	REPL^c^ (mean)	MLST(ST)
				1	2				
1	CRC1	A or C	RIF, TMP	−	+	1.72	0.63	320.62	−
2	CRC1	B2	CIP, SXT, CLR, RIF, TMP	+	−	2.34	0.2	427.86	−
10	CRC1	B2	TET, CIP, SXT, CLR, RIF, TMP	+	−	2.49	0.22	519.55	131
11	CRC1	B2	CLR, RIF, TMP	+	−	1.55	0.45	163.72	167
12	CRC1	B2	TET, CIP, CLR, RIF,	+	+	2.07	0.22	244.37	−
14	CRC1	D	TET, CIP, SXT, CLR, RIF, TMP	+	−	1.53	0.47	116.28	−
15	CRC1	D	CIP, SXT, CLR, RIF, TMP	+	+	1.62	0.19	338.00	−
40	CRC2	B2	TET, SXT, CLR, RIF, TMP	−	+	2.16	0.47	645.85	135
32	CRC2	D	TET, CLR, Rif, TMP	+	−	1.92	0.41	322.21	−
44	CRC2	B1	TET, SXT, CLR, RIF, TMP	+	+	1.34	0.69	492.00	−
45	CRC2	B2	TET, RIF,	−	−	2.07	0.37	394.48	−
46	CRC2	B2	CIP, CLR, RIF,	+	−	1.67	0.7	387.45	131
48	CRC2	B2	CLR, RIF,	−	−	3.81	0.68	478.67	14
49	CRC2	D	CLR, RIF	−	−	2.73	0.6	881.37	95
51	CRC2	E	TET, SXT, CLR, RIF, TMP	+	+	1.41	0.39	114.16	−
71	CRChis	B2	CIP, CLR, RIF,	+	−	1.41	0.16	119.53	−
72	CRChis	B2	SXT, CLR, RIF, TMP	−	+	1.55	0.18	187.05	1,193
73	CRChis	D	SXT, CLR, RIF, TMP	+	−	1.38	0.38	116.27	−
74	CRChis	D	CLR, RIF, TMP	+	−	1.02	0.24	187.00	−
101	FH	D	CLR, RIF	−	−	1.72	0.13	138.70	73
104	FH	E	CLR	−	−	1.38	0.13	176.37	−
131	Control	B2	CLR, RIF	−	−	1.00	0.11	128.33	167
132	Control	B2	CLR, RIF	−	−	1.05	0.37	113.69	837
133	Control	B2	SXT, CLR, RIF, TMP	+	+	1.10	0.14	137.65	−

RIF, Rifampicin; TMP, Trimethoprim; CIP, Ciprofloxacin; CLR, Clarithromycin; SXT, Trimethoprim / Sulfamethoxazole; TET, Tetracycline. CRC1, colorectal cancer in situ (Tis); CRC2, CRC with tumor stage T1, T2, or T3; CRChis, with CRC history; FH, with a family history of CRC. ^a^Grade of adhesion. ^b^Percentage of inoculum surviving after 1 h of gentamicin treatment (number of intracellular bacteria/initial inoculum × 100). ^c^Number of intracellular bacteria at 24 h post-infection/number of bacteria at 1 h post-infection × 100.

Clonal relationship analysis of isolates showed that ST131 and ST838 belonged to CC131, ST1193, and ST14 belonged to CC14, ST167 belonged to CC10, ST73 belonged to CC73, and ST95 belonged to CC95. ST135 was singleton. ST838 is a DLV from ST131, which differs in two alleles of mdh and fumC, and ST1193 is a SLV from ST14, which differs in the icd allele.

**Figure 1:**
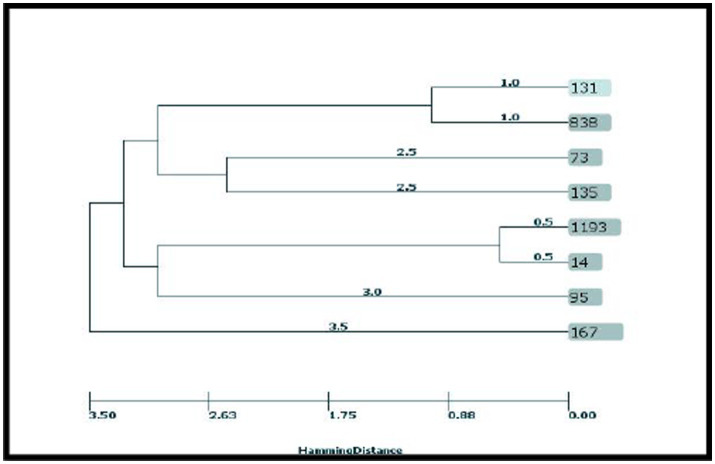
UPGMA dendrogram of AIEC clinical isolates.

**Figure 2:**
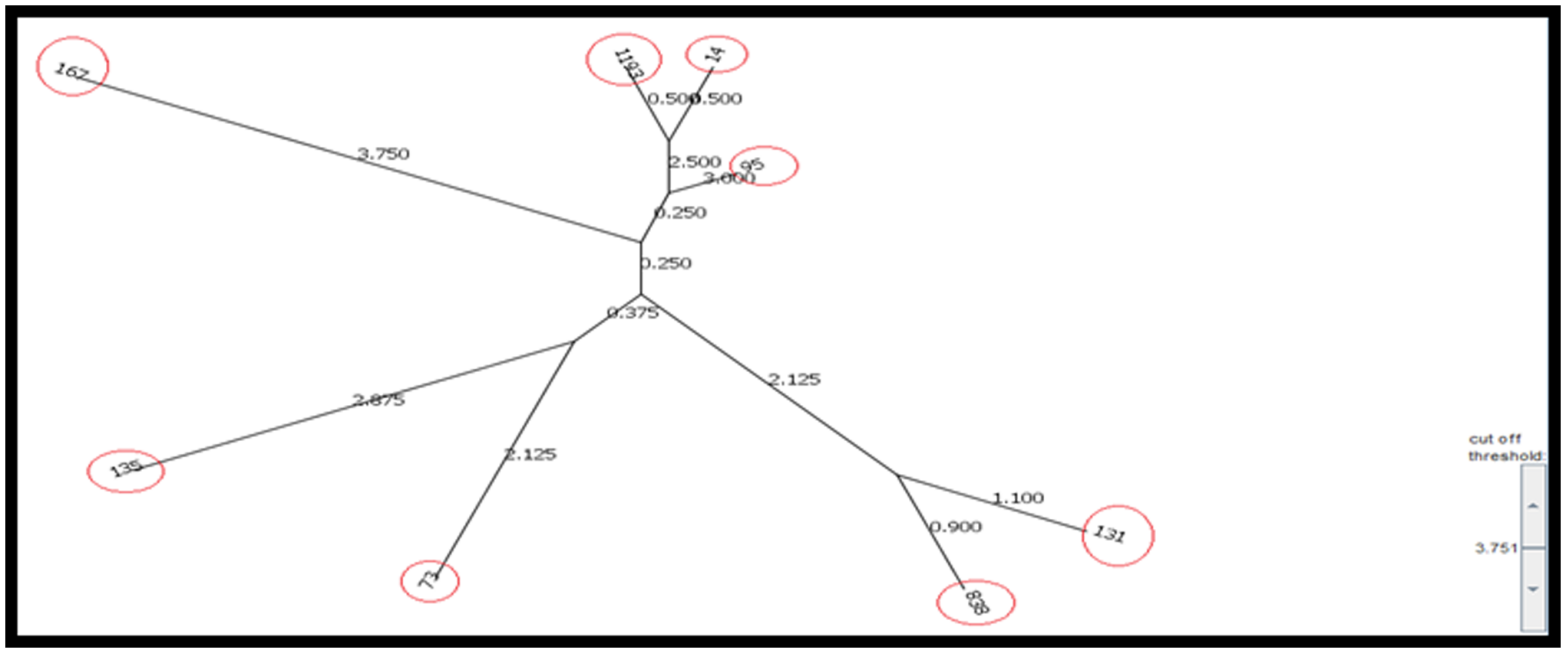
Position of STs found in the study using neighbor-joining Saitou-Nei Criterion.

According to the results, the STs obtained from the CRC2 group were ST131, ST14, ST135, and ST95. ST131 and ST167 were obtained from the CRC1 group. ST1193 and ST73 were from CRchis and FH groups, respectively, and the STs obtained from the control group were ST838 and ST167 (Supplementary Table S4). The phylogeny tree and kinship of the isolates are shown in Figures 1, 2.

## 4 Discussion

The results of the present study revealed that the frequency of AIEC isolates was notably higher in both groups of CRC patients, particularly among those with tumor stages 1–3 (CRC2), with a frequency of 33.4% compared to 12.5% in the control group. The highest antibiotic resistance of AIEC isolates was seen in rifampin and trimethoprim, and the highest antibiotic sensitivity was seen in trimethoprim-sulfamethoxazole. Also, a notable association was observed between the presence of integron 1 and the invasion, survival, and replication capabilities within macrophages among AIEC strains. According to the MLST results, the most known sequence type of ST131 belonged to CC131 containing integron 1.

CRC is a multifactorial disease influenced by various factors, each contributing differently to the occurrence and formation of the tumor, among which the composition of the gastrointestinal microbiome plays a significant role. Within the gastrointestinal microbiome strains, the *E. coli* AIEC strain stands out as a potential contributor to tumor development through its diverse capabilities, including intracellular invasion and proliferation, along with the production of carcinogenic toxins (Martinez-Medina et al., 2020). Also, strains of this bacteria exhibiting antibiotic resistance pose a potential health risk for individuals who are infected with this pathotype. Therefore, it seems necessary to determine the antibiotic resistance profile of AIEC isolates to propose a suitable treatment model for the treatment of patients who are colonized with this pathotype.

The results of this study showed that more tumors were found in the distal colon (65%) than in the proximal colon (35%). This finding was in line with the results of a previous study conducted by Iyadorai et al. (2020) who reported that 58.3% of CRC patients had distal colon tumors and 41.66% of them had proximal colon tumors. According to a meta-analysis by Wang et al., in Asian countries, the frequency of distal and proximal colon tumors was 45.2% and 39.3%, respectively (Wong et al., 2019). Our results also showed a higher proportion of intracellular and pathovar AIEC strains among the CRC patient groups. Also, our results revealed a higher proportion of invasion and replication survival within the macrophages of AIEC isolates of CRC patients in the T1–T3 stage compared to those from healthy individuals. Furthermore, our findings revealed a higher proportion of invasion and replication survival within macrophages among AIEC isolates from CRC patients in tumor stages T1–T3 than those of healthy subjects. In a study by Lee et al. (2019), the frequency of AIEC pathovars was 44.4 and 28% in patients with ulcerative colitis (UC) and Crohn’s disease, respectively, and 28.6% in the healthy control subjects. In their study, AIEC isolates from IBD patients exhibited notable distinctions compared to those from the control group in terms of attachment, invasion, survival, and replication rates within macrophages. In a recent study by Siles et al., the reported frequency of AIEC pathovars was 6.7% and 35.7% in CRC and UC patients, respectively (López-Siles et al., 2022). Furthermore, survival and replication within macrophages in the isolates of UC patients were higher compared to those from CRC patients, whereas the invasion in the isolates of CRC patients surpassed that of both UC patients and the control groups. Therefore, further comprehensive studies are needed to elucidate the essential virulence factors crucial in the cellular pathogenesis of diverse clinical conditions. Adherence of AIEC pathovar to intestinal epithelial cells is partially mediated by type 1 pili with carcinoembryonic glycoprotein 6 according to a mannose-dependent pattern. Carcinoembryonic6 is overexpressed in CRC patients. This disease makes people susceptible to AIEC over-colonization (Martinez-Medina and Garcia-Gil, 2014). The heightened colonization potential of this pathovar in CRC patients may result in increased levels of pro-inflammatory cytokine IL-8 and chemokine CCL20 in large intestinal epithelial cells, which can induce the recruitment of macrophages and dendritic cells to the infection site (Subramanian et al., 2008). These strains possess the capability to initiate internalization into macrophages and survive there without inducing host cell death. This feature allows the bacteria to pass through the human intestinal barrier to deeper tissues to continuously activate macrophages and induce granuloma formation, which is the precursor for malignancy (Chervy et al., 2020). Macrophages infected with AIEC are known to secrete large amounts of TNFα. These strains possess the ability to initiate internalization into macrophages and survive inside the macrophage without inducing host cell death. This feature enables the bacteria to pass through human intestinal barriers, penetrate deeper tissues, continuously activate macrophages, and induce granuloma production, which serves as a precursor to malignancy (Glasser et al., 2001). In other words, both the presence of this bacterium and CRC act as contributing factors to the progression of the carcinogenic cycle effect of these *E. coli* pathovars with their synergistic effect. Meanwhile, several studies have shown the role of AIEC isolates as potential contributors to the pathogenesis of CRC (Bonnet et al., 2014). Swidsinski et al. (1998)and Martin et al. (2004) have reported an increase in the frequency of AIEC strains ranging from 33% to 80% in CRC patients compared to the control group (range, less than 1% to 9%). The characteristic feature of AIEC isolates is the ability to invade and survive intracellularly. Therefore, it is imperative to assess the antibiotic resistance profile of AIEC isolates against antibiotics with intracellular penetration potential to devise effective strategies for the prevention and treatment of patients colonized with these pathogenic strains (Buc et al., 2013). Regarding the antibiotic resistance profile of AIEC and non-AIEC intracellular isolates to antibiotics effective on intracellular bacteria; trimethoprim-sulfamethoxazole, trimethoprim, ciprofloxacin, and tetracycline, the highest antibiotic resistance of AIEC isolates was to rifampin and trimethoprim, and the highest antibiotic sensitivity was to trimethoprim-sulfamethoxazole. In the limited body of literature examining the antibiotic resistance of AIEC isolates, the study conducted by Martinez-Medina et al. documented the highest resistance rate to tetracycline at 31.8%, while resistance to trimethoprim-sulfamethoxazole was observed in only 9.1% of cases. Conversely, among non-AIEC bacteria, the highest antibiotic resistance was reported against tetracycline (29.6%), followed by trimethoprim-sulfamethoxazole (14.8%; López-Siles et al., 2022). In the study by Dogan et al. (2012), focusing on intracellular *E. coli* isolates isolated from Crohn’s patients, the highest antibiotic resistance was reported to tetracycline and clarithromycin (38%), followed by trimethoprim-sulfamethoxazole and ciprofloxacin (29%). Contrary to our results and those of other studies, the study conducted by Subramanian et al. (2008) and Elliott et al. (2013) did not find a significant prevalence of antibiotic resistance among AIEC isolated from patients with IBD. Clinically, the acquisition of drug-resistance genes through horizontal gene transfer holds greater significance. The presence of integrons, mobile genetic elements harboring various resistance genes, represents one of the mechanisms of acquiring antibiotic resistance through the horizontal transfer of genes (Shetty et al., 2023).

In the present study, molecular analyses of genomic islands carrying antibiotic resistance genes revealed an elevated frequency of integron 1 and 2 in AIEC strains compared to non-AIEC strains. Specifically, 79.2% and 45.8% of AIEC isolates harbored integron 1 and integron 2, respectively, with both integron 1 and integron 2 being concurrently present in 41.6% of AIEC isolates. Furthermore, the frequency of integrons 1 and 2 in non-AIEC isolates was 59.2% and 61.2%, respectively, and both integron 1 and integron 2 genes were identified in 38% of non-AIEC isolates. Class 1 integron is often carried on plasmids or transposons, transports several antibiotic resistance genes, and is considered one of the antibiotic resistance gene propagation machines (Behera et al., 2023). In this study, the isolates possessing integron 1 and 2 exhibited the highest resistance to rifampin followed by trimethoprim. Further, a notable association between the presence of integron 1, the percentage of invasion (*p* = 0.03), and the survival and replication within macrophages (*p* = 0.004) was found in AIEC strains. Therefore, it can be suggested the emergence of hazardous strains within the population of CRC patients, is characterized by elevated antibiotic resistance alongside virulence factors. In the study by Medina et al., most of the AIEC strains with the sulfonamide resistance gene (*sul-1*) were found to harbor integron 1 (Martinez-Medina et al., 2020). Class 1 integron is commonly situated on plasmids or transposons, where it coexists with resistance genes, playing a pivotal role in the dissemination of antibiotic resistance. In a study focusing on strains such as AIEC—UPEC tosA^+^, the heightened antibiotic resistance was attributed to the presence of integrons, and it was suggested that their virulence is related to the presence of core-associated genes (*fimH*, *csgA*, *motAB*, and *fliC*), as well as the genes linked to pathogenicity islands (PAIs-associated genes; Xicohtencatl-Cortes et al., 2019). Consequently, regarding AIEC pathovars, it seems to be necessary to investigate the presence of pathogenicity islands in strains exhibiting elevated antibiotic resistance and virulence factors to mitigate the dissemination of these strains. Considering reports highlighting the involvement of AIEC strains in various gastrointestinal diseases including CRC, therefore, the occurrence of antibiotic resistance in these isolates is considered a serious public health threat (Kaushik et al., 2018).

A strong association between the extent of antibiotic resistance and integrons has been recorded in clinical isolates from different geographical locations. This underscores the pivotal role of these genetic elements in facilitating the dissemination of antibiotic resistance determinants. Discrepancies observed in the prevalence of integrons and antibiotic resistance in various studies could be attributed to the difference in geographical location, the origin of infection and its distribution, techniques employed for integron detection, as well as the dosage regimens of antibiotics administered. Although more studies are needed to evaluate the antibiotic resistance profile of AIEC isolates against intracellular antibiotics, it is worth considering that in the current study area, the prevalent utilization of antibiotics in human medicine for therapeutic and prophylactic purposes may contribute to the emergence of strains resistant to antibiotics effective against AIEC intracellular bacteria. Bacteria can acquire the antibiotic resistance phenotype through the horizontal transfer of genes or the occurrence of a *de novo* mutation in the bacterial genome (Ramatla et al., 2023).

Based on the epidemiological analysis conducted by MLST, since 2000, ST131 emerged as an epidemic and pandemic clone with elevated risk and in most cases, high antibiotic resistance (Riley, 2014). ST131 strains demonstrate greater virulence compared to other AIEC clonals. Due to a combination of high antibiotic resistance and high virulence characteristics, ST131 strains have a competitive advantage over other categories, facilitating their proliferation across various environments with different features (Blanco et al., 2011).

Based on studies *E. coli* ST131 is an extensively reported MDR ExPEC lineage associated with both UTI and BSI (Boll et al., 2020). Many typical ExPEC-associated virulence factors, including P fimbriae, hemolysins, and factors conferring increased serum survival and iron uptake, have been identified in ST131 isolates (Blanco et al., 2013; Olesen et al., 2014), but overall little is known about which genes (whether they promote virulence or other phenotypes) have made this clonal lineage so successful. In contrast, virulence traits associated with diarrheagenic *E. coli* (DEC) pathotypes have thus far largely been absent from ST131 isolates (Olesen et al., 2014). This plasticity includes frequent recombination and plasmid flux, particularly involving IncF-type plasmids, which facilitates the spread of antibiotic resistance and virulence genes (Fernández Lanza et al., 2014; Stoesser et al., 2016). Importantly, in ST131, compensatory mutations offset the fitness costs that result from these genomic events (McNally et al., 2013). In this study, we have reported ST131 from patients with CRC, considering that these strains belong to phylogroup B2 and show high antibiotic resistance, the isolation of AIEC strains from patients with malignancy needs attention and follow-up and is important.

Given the high prevalence of strain, identifying its transmission and distribution methods in the studied population is of particular importance (Doumith et al., 2015; Namaei et al., 2017). In the current study, a collective 30% of the isolates were classified under clonal complex 131 and phylogeny group B2. Among these, 20% of the isolates were attributed to ST131, all of which harbored the integron 1 gene. Additionally, 10% of the isolates were categorized as ST838, originating from the control group and lacking integrons.

In the study by Martinez-Medina et al. (2009), 40% of intestinal isolates and 25% of extraintestinal isolates belonged to ST131, all of which were within phylogeny group B2 and serotype O25:H4.

In a study conducted in China, 10.17% of isolates identified as ST131, and their virulence score was significantly higher compared to non-131 isolates (Liu et al., 2022). In the research conducted by Lee et al., which focused on carbapenem-resistant *E. coli* isolated from feces, it was found that the selected isolates all belonged to ST 410. Comparative genomic analysis of ST167, a member of clonal complex 10, unveiled a complete absence of the wca operon. This operon is responsible for cholanic acid biosynthesis within the LPS biosynthesis pathway. The absence of this operon results in the production of a short-chain O antigen, thereby facilitating successful colonization and evasion from the immune system (Zeng et al., 2019).

Recent research has indicated that the O89b antigen is a new variant of the O antigen present in all ST167 strains (Zeng et al., 2019). In this clade, *Klebsiella pneumoniae* K48 capsular synthesis gene cluster have also been detected and a majority of the strains carrying NDM-5 have been identified. Consequently, the combined presence of antibiotic resistance and virulence factors poses a potential risk for the global dissemination of this particular sequence type. In the present study, ST167 emerged as another prevalent clade, that was isolated from a low-grade dysplasia (CRC1) patient who carried integron I and a participant in the control group without integron, constituting 20% of the isolates. Both instances were categorized under phylogeny group B2. Solegi et al. highlighted ST167 as one of the most recurrent sequence types (11.5%) among clinical *E. coli* isolates. In this study, this ST showed a high frequency of drug resistance genes and integron 1 (Solgi et al., 2017).

The third most common clonal complex among AIEC isolates in this study was clonal complex 14, all of which belong to phylogenetic group B2. ST14 was isolated from a patient with tumor stage T2, devoid of integrons, whereas ST1193 was isolated from a patient with a history of colorectal cancer (CRChis) who had integron II. ST1193 has recently attracted much attention worldwide as a fluoroquinolone-resistant clonal group of extraintestinal infections (Johnson et al., 2019). These isolates are derived from clonal complex 14 and, like ST131, mainly belong to pathogenic phylogenetic group B2. This ST exhibits a notable mutagenic ability and also shows strong biofilm formation ability (Crémet et al., 2013). In addition, these isolates have been isolated worldwide from many human isolates from Australia (Platell et al., 2012), China (Zhao et al., 2015; Lao et al., 2017; Wu et al., 2017), South Korea (Chang et al., 2014), Norway (Jørgensen et al., 2017), and America (Tchesnokova et al., 2019), and most of the isolates were separated from fecal or urinary samples.

In this study, ST95 was isolated from a patient with T3 invasion depth (CRC2), which belonged to phylogeny group D and did not have integron. Notably, among the studied AIEC isolates, this particular strain displayed the highest binding percentage (2.73%), invasion (0.6%) as well as survival and replication (881.37%) within macrophages. In a study conducted by Petersen et al. (2009) in Denmark on IBD isolates, ST95 was detected in 7.7% of *E. coli* isolates. It’s worth noting that certain human infections, both gastrointestinal and extraintestinal, can have origins in animals and can be transmitted through contaminated meat. *Escherichia coli* strain ST95 is one of these strains that is commonly identified in many human infections, serving as a dominant STs in causing avian colibacillosis (Manges, 2016; Poulsen et al., 2017). Studies show a strong association between avian *E. coli* infections and human extraintestinal *E. coli* infections, and these two strains have shown great similarities in PFGE studies (Moulin-Schouleur et al., 2007; Pires-dos-Santos et al., 2013). Furthermore, whole-genome sequencing studies have shown close relationships between these two strains (Manges, 2016; Poulsen et al., 2017). Consequently, there is growing speculation among researchers regarding the potential zoonotic nature of this infection. In the present study, ST73 was identified as a participant in the FH group. This strain belonged to phylogeny group D and did not exhibit integron presence. Another study has highlighted ST73 as the most prevalent sequence type in IBD patient samples and it was detected in 15.4% of *E. coli* isolates. In a study in Spain (2009), four AIEC isolates from extraintestinal infection and five AIEC isolates from intestinal infection were typed by the MLST method. This study revealed that two isolates from Crohn’s patients, one from urinary tract infection, and one from septic infection belonged to ST73, all of which belonged to phylogroup B2. Additionally, one isolate from Crohn’s patient and another from septicemia were classified as ST135. Finally, two ulcerative colitis patients, one from the control group, and one isolated from urinary infection were categorized as ST13, all within phylogeny group B2. Flament-Simon et al. conducted a study on clinical *E. coli* isolates in France in 2019, revealing the prevalence of various sequence types (STs) including ST131 (26.4%), ST73 (5.6%), ST88 (4.3%), ST10 (4.3%), ST141 (4.1%), ST69 (3.8%), and ST95 (3/6%). In our study, ST135 was identified in a patient with high-grade dysplasia (CRC2), falling under phylogeny group B2, and harboring integron 2.

## 5 Conclusion

The present study revealed a heightened frequency of AIEC isolates in the biopsies of patients with CRC and those with a previous history of this disease compared to the control group and subjects with a family history of this disease. Although CRC patient isolates exhibited elevated levels of attachment, invasion, survival, and replication within macrophages compared to the other groups, no significant association was observed with the antibiotic resistance profile of these isolates.

AIEC isolates exhibited pronounced resistance to intracellular antibiotics, particularly surpassing a threshold of 2 μg/mL for rifampin. Integron 1 was notably more prevalent among AIEC isolates compared to integron 2, with approximately 40% of isolates harboring both integrons concurrently. Based on the results of MLST, the distribution pattern of STs is different among the studied groups. Also, the presence of pandemic STs underscores the significance of screening and managing patients afflicted with these isolates. Additionally, comprehensive prospective clinical investigations are warranted to evaluate whether the identification of this pathovar could serve as a prognostic indicator for CRC and therapeutic modalities specifically targeting drug-resistant AIECs, such as phage therapy, bacteriocins, and anti-adhesion compounds, could offer efficacy in the prevention and treatment of CRC.

## Data availability statement

The original contributions presented in the study are included in the article/Supplementary material, further inquiries can be directed to the corresponding author.

## Ethics statement

The studies involving humans were approved by Working Group of Research Ethics Committee of Isfahan University of Medical Sciences—Al-Zahra Research Centers Group. The studies were conducted in accordance with the local legislation and institutional requirements. The participants provided their written informed consent to participate in this study.

## Author contributions

AH: Data curation, Formal analysis, Investigation, Methodology, Project administration, Resources, Writing – original draft, Writing – review & editing. ME: Conceptualization, Funding acquisition, Project administration, Supervision, Validation, Writing – review & editing. FM: Conceptualization, Project administration, Supervision, Validation, Visualization, Writing – original draft, Writing – review & editing. SM: Conceptualization, Data curation, Formal analysis, Investigation, Methodology, Writing – review & editing. PK: Methodology, Software, Writing – review & editing. TS: Validation, Visualization, Writing – review & editing. AF: Conceptualization, Data curation, Writing – review & editing. RK: Formal analysis, Investigation, Methodology, Software, Supervision, Validation, Visualization, Writing – original draft, Writing – review & editing.

## Footnotes

^1^http://mlst.warwick.ac.uk/mlst/dbs/*E.coli*

## References

[ref1] Behera M. Parmanand S. Roshan M. Rajput S. Gautam D. Vats A.. (2023). Novel aadA5 and dfrA17 variants of class 1 integron in multidrug-resistant *Escherichia coli* causing bovine mastitis. Appl. Microbiol. Biotechnol. 107, 433–446. doi: 10.1007/s00253-022-12304-3, PMID: 36454252

[ref2] Bhat B. A. Mir R. A. Qadri H. Dhiman R. Almilaibary A. Alkhanani M.. (2023). Integrons in the development of antimicrobial resistance: critical review and perspectives. Front. Microbiol. 14:1231938. doi: 10.3389/fmicb.2023.1231938, PMID: 37720149 PMC10500605

[ref3] Blanco J. Mora A. Mamani R. López C. Blanco M. Dahbi G.. (2011). National survey of *Escherichia coli* causing extraintestinal infections reveals the spread of drug-resistant clonal groups O25b: H4-B2-ST131, O15: H1-D-ST393 and CGA-D-ST69 with high virulence gene content in Spain. J. Antimicrob. Chemother. 66, 2011–2021. doi: 10.1093/jac/dkr235, PMID: 21669946

[ref4] Blanco J. Mora A. Mamani R. López C. Blanco M. Dahbi G.. (2013). Four main virotypes among extended-spectrum-β-lactamase-producing isolates of *Escherichia coli* O25b: H4-B2-ST131: bacterial, epidemiological, and clinical characteristics. J. Clin. Microbiol. 51, 3358–3367. doi: 10.1128/JCM.01555-13, PMID: 23926164 PMC3811668

[ref5] Boll E. J. Overballe-Petersen S. Hasman H. Roer L. Ng K. Scheutz F.. (2020). Emergence of enteroaggregative *Escherichia coli* within the ST131 lineage as a cause of extraintestinal infections. MBio 11, 00353–00320. doi: 10.1128/mBio.00353-20 PMC7240153 32430467

[ref6] Bonnet M. Buc E. Sauvanet P. Darcha C. Dubois D. Pereira B.. (2014). Colonization of the human gut by E. Coli and colorectal cancer risk. Clin. Cancer Res. 20, 859–867. doi: 10.1158/1078-0432.CCR-13-1343, PMID: 24334760

[ref7] Buc E. Dubois D. Sauvanet P. Raisch J. Delmas J. Darfeuille-Michaud A.. (2013). High prevalence of mucosa-associated *E. coli* producing cyclomodulin and genotoxin in colon cancer. PLoS One 8:e56964. doi: 10.1371/journal.pone.0056964, PMID: 23457644 PMC3572998

[ref8] Camprubí-Font C. Ewers C. Lopez-Siles M. Martinez-Medina M. (2019). Genetic and phenotypic features to screen for putative adherent-invasive *Escherichia coli*. Front. Microbiol. 10:108. doi: 10.3389/fmicb.2019.00108, PMID: 30846972 PMC6393329

[ref9] Chang J. Yu J. Lee H. Ryu H. Park K. Park Y.-J. (2014). Prevalence and characteristics of lactose non-fermenting *Escherichia coli* in urinary isolates. J. Infect. Chemother. 20, 738–740. doi: 10.1016/j.jiac.2014.07.005, PMID: 25193040

[ref10] Chen W.-p. Kuo T.-T. (1993). A simple and rapid method for the preparation of gram-negative bacterial genomic DNA. Nucleic Acids Res. 21:2260. doi: 10.1093/nar/21.9.2260, PMID: 8502576 PMC309503

[ref11] Chervy M. Barnich N. Denizot J. (2020). Adherent-Invasive *E. coli*: update on the lifestyle of a troublemaker in Crohn’s disease. Int. J. Mol. Sci. 21:3734. doi: 10.3390/ijms21103734 32466328 PMC7279240

[ref12] Cho Y. H. Renouf M. J. Omotoso O. McPhee J. B. (2022). Inflammatory bowel disease-associated adherent-invasive *Escherichia coli* have elevated host-defense peptide resistance. FEMS Microbiol. Lett. 369:fnac098. doi: 10.1093/femsle/fnac098, PMID: 36208952

[ref13] Crémet L. Caroff N. Giraudeau C. Reynaud A. Caillon J. Corvec S. (2013). Detection of clonally related *Escherichia coli* isolates producing different CMY β-lactamases from a cystic fibrosis patient. J. Antimicrob. Chemother. 68, 1032–1035. doi: 10.1093/jac/dks520, PMID: 23302581

[ref14] Dalmasso G. Nguyen H. T. Faïs T. Massier S. Barnich N. Delmas J.. (2019). Crohn’s disease-associated adherent-invasive *Escherichia coli* manipulate host autophagy by impairing SUMOylation. Cells 8:35. doi: 10.3390/cells8010035, PMID: 30634511 PMC6357120

[ref15] Deng Y. Bao X. Ji L. Chen L. Liu J. Miao J.. (2015). Resistance integrons: class 1, 2 and 3 integrons. Ann. Clin. Microbiol. Antimicrob. 14, 1–11. doi: 10.1186/s12941-015-0100-6 26487554 PMC4618277

[ref16] Dogan B. Scherl E. Bosworth B. Yantiss R. Altier C. McDonough P. L.. (2012). Multidrug resistance is common in *Escherichia coli* associated with ileal Crohn's disease. Inflamm. Bowel Dis. 19, 141–150. doi: 10.1002/ibd.22971 22508665

[ref17] Doumith M. Day M. Ciesielczuk H. Hope R. Underwood A. Reynolds R.. (2015). Rapid identification of major *Escherichia coli* sequence types causing urinary tract and bloodstream infections. J. Clin. Microbiol. 53, 160–166. doi: 10.1128/JCM.02562-14, PMID: 25355761 PMC4290915

[ref18] Elliott T. R. Hudspith B. N. Wu G. Cooley M. Parkes G. Quiñones B.. (2013). Quantification and characterization of mucosa-associated and intracellular *Escherichia coli* in inflammatory bowel disease. Inflamm. Bowel Dis. 19, 2326–2338. doi: 10.1097/MIB.0b013e3182a38a92, PMID: 23989750

[ref19] Fayyazi A. Halaji M. Sadeghi A. Havaei S. A. (2020). High frequency of integrons and efflux pump in uropathogenic *Escherichia coli* isolated from Iranian kidney and non-kidney transplant patients. Gene Rep. 21:100873. doi: 10.1016/j.genrep.2020.100873

[ref20] Fazeli H. Hasan Emami S. M. (2021). Compare characteristics of mucosa-associated adherent-invasive *Escherichia coli* isolated from colorectal cancer patients. Iran J Gastroenterol Hepatol 26.

[ref21] Fedorak R. N. Ismond K. P. (2016). Practical considerations and the intestinal microbiome in disease: antibiotics for IBD therapy. Dig. Dis. 34, 112–121. doi: 10.1159/000443014, PMID: 26982586

[ref22] Fernández Lanza V. Md T. H. MdP G. B. Mora A. Blanco J. Coque T. M.. (2014). Plasmid flux in *Escherichia coli* ST131 sublineages, analyzed by plasmid constellation network (PLACNET), a new method for plasmid reconstruction from whole genome sequences. PLoS Genet. 10:e1004766. doi: 10.1371/journal.pgen.1004766 25522143 PMC4270462

[ref23] Glasser A.-L. Boudeau J. Barnich N. Perruchot M.-H. Colombel J.-F. Darfeuille-Michaud A. (2001). Adherent invasive *Escherichia coli* strains from patients with Crohn's disease survive and replicate within macrophages without inducing host cell death. Infect. Immun. 69, 5529–5537. doi: 10.1128/IAI.69.9.5529-5537.2001, PMID: 11500426 PMC98666

[ref24] Halaji M. Feizi A. Mirzaei A. Sedigh Ebrahim-Saraie H. Fayyazi A. Ashraf A.. (2020). The global prevalence of class 1 integron and associated antibiotic resistance in *Escherichia coli* from patients with urinary tract infections, a systematic review and meta-analysis. Microb. Drug Resist. 26, 1208–1218. doi: 10.1089/mdr.2019.0467, PMID: 32282274

[ref25] Iyadorai T. Mariappan V. Vellasamy K. M. Wanyiri J. W. Roslani A. C. Lee G. K.. (2020). Prevalence and association of pks+ *Escherichia coli* with colorectal cancer in patients at the university Malaya medical Centre, Malaysia. PLoS One 15:e0228217. doi: 10.1371/journal.pone.0228217, PMID: 31990962 PMC6986756

[ref26] Joddha H. B. Mathakiya R. A. Joshi K. V. Khant R. B. Golaviya A. V. Hinsu A. T.. (2023). Profiling of antimicrobial resistance genes and Integron from *Escherichia coli* isolates using whole genome sequencing. Genes. 14:1212. doi: 10.3390/genes14061212, PMID: 37372392 PMC10298372

[ref27] Johnson T. J. Elnekave E. Miller E. A. Munoz-Aguayo J. Flores Figueroa C. Johnston B.. (2019). Phylogenomic analysis of extraintestinal pathogenic *Escherichia coli* sequence type 1193, an emerging multidrug-resistant clonal group. Antimicrob. Agents Chemother. 63, 01913–01918. doi: 10.1128/AAC.01913-18 PMC6325179 30348668

[ref28] Jørgensen S. Sunde M. Fladberg Ø. Leegaard T. Berg E. Steinbakk M., (2017). Fluoroquinolone resistant Escherichia coli ST1193-another global successful clone. In: *27th European Congress of Clinical Microbiology and Infectious Diseases (ECCMID)*, P0204.

[ref29] Kamali Dolatabadi R. Fazeli H. Emami M. H. Karbasizade V. Maghool F. Fahim A.. (2022). Phenotypicand genotypic characterization of clinical isolates of intracellular adherent–invasive *Escherichia coli* among different stages, family history, and treated colorectal cancer patients in Iran. Front. Cell. Infect. Microbiol. 12:938477. doi: 10.3389/fcimb.2022.938477, PMID: 35899040 PMC9309365

[ref30] Kaushik M. Kumar S. Kapoor R. K. Virdi J. S. Gulati P. (2018). Integrons in Enterobacteriaceae: diversity, distribution and epidemiology. Int. J. Antimicrob. Agents 51, 167–176. doi: 10.1016/j.ijantimicag.2017.10.004, PMID: 29038087

[ref31] Lao J. Chen Y. Li Z.-C. Li Q. Zhang J. Liu J.. (2017). A deep learning-based radiomics model for prediction of survival in glioblastoma multiforme. Sci. Rep. 7:10353. doi: 10.1038/s41598-017-10649-8, PMID: 28871110 PMC5583361

[ref32] Lee J. G. Han D. S. Jo S. V. Lee A. R. Park C. H. Eun C. S.. (2019). Characteristics and pathogenic role of adherent-invasive *Escherichia coli* in inflammatory bowel disease: potential impact on clinical outcomes. PLoS One 14:e0216165. doi: 10.1371/journal.pone.0216165, PMID: 31034508 PMC6488085

[ref33] Liu X. Li X. Yang A.-w. Tang B. Jian Z.-j. Zhong Y.-m.. (2022). Community fecal carriage and molecular epidemiology of extended-spectrum β-lactamase-and carbapenemase-producing *Escherichia coli* from healthy children in the central South China. Infect Drug Resist. 15, 1601–1611. doi: 10.2147/IDR.S357090, PMID: 35418762 PMC8995156

[ref34] López-Siles M. Camprubí-Font C. Gómez del Pulgar E. M. Sabat Mir M. Busquets D. Sanz Y.. (2022). Prevalence, abundance, and virulence of adherent-invasive *Escherichia coli* in ulcerative colitis, colorectal cancer, and coeliac disease. Front. Immunol. 13:569. doi: 10.3389/fimmu.2022.748839 PMC8960851 35359974

[ref35] Manges A. (2016). Escherichia coli and urinary tract infections: the role of poultry-meat. Clin. Microbiol. Infect. 22, 122–129. doi: 10.1016/j.cmi.2015.11.010, PMID: 26679924

[ref36] Martin H. M. Campbell B. J. Hart C. A. Mpofu C. Nayar M. Singh R.. (2004). Enhanced *Escherichia coli* adherence and invasion in Crohn’s disease and colon cancer. Gastroenterology 127, 80–93. doi: 10.1053/j.gastro.2004.03.054, PMID: 15236175

[ref37] Martinez-Medina M. Garcia-Gil L. J. (2014). *Escherichia coli* in chronic inflammatory bowel diseases: an update on adherent invasive *Escherichia coli* pathogenicity. World J. Gastrointest. Pathophysiol. 5, 213–227. doi: 10.4291/wjgp.v5.i3.213, PMID: 25133024 PMC4133521

[ref38] Martinez-Medina M. Mora A. Blanco M. López C. Alonso M. P. Bonacorsi S.. (2009). Similarity and divergence among adherent-invasive Escherichia coli and extraintestinal pathogenic *E. coli* strains. J. Clin. Microbiol. 47, 3968–3979. doi: 10.1128/JCM.01484-09, PMID: 19828750 PMC2786640

[ref39] Martinez-Medina M. Strozzi F. Ruiz Del Castillo B. Serrano-Morillas N. Ferrer Bustins N. Martínez-Martínez L. (2020). Antimicrobial resistance profiles of adherent invasive *escherichia coli* show increased resistance to β-lactams. Antibiotics. 9:251. doi: 10.3390/antibiotics9050251, PMID: 32414140 PMC7277491

[ref40] McNally A. Alhashash F. Collins M. Alqasim A. Paszckiewicz K. Weston V.. (2013). Genomic analysis of extra-intestinal pathogenic *Escherichia coli* urosepsis. Clin. Microbiol. Infect. 19, e328–e334. doi: 10.1111/1469-0691.12202, PMID: 23573792

[ref41] Moulin-Schouleur M. Répérant M. Laurent S. Brée A. Mignon-Grasteau S. Germon P.. (2007). Extraintestinal pathogenic *Escherichia coli* strains of avian and human origin: link between phylogenetic relationships and common virulence patterns. J. Clin. Microbiol. 45, 3366–3376. doi: 10.1128/JCM.00037-07, PMID: 17652485 PMC2045314

[ref42] Mousavifar L. Touaibia M. Roy R. (2018). Development of mannopyranoside therapeutics against adherent-invasive *Escherichia coli* infections. Acc. Chem. Res. 51, 2937–2948. doi: 10.1021/acs.accounts.8b00397, PMID: 30289687

[ref43] Namaei M. H. Yousefi M. Ziaee M. Salehabadi A. Ghannadkafi M. Amini E.. (2017). First report of prevalence of CTX-M-15-producing *Escherichia coli* O25b/ST131 from Iran. Microb. Drug Resist. 23, 879–884. doi: 10.1089/mdr.2016.0272, PMID: 28437226 PMC5665095

[ref44] Olesen B. Frimodt-Møller J. Leihof R. F. Struve C. Johnston B. Hansen D. S.. (2014). Temporal trends in antimicrobial resistance and virulence-associated traits within the *Escherichia coli* sequence type 131 clonal group and its H 30 and H 30-Rx subclones, 1968 to 2012. Antimicrob. Agents Chemother. 58, 6886–6895. doi: 10.1128/AAC.03679-14, PMID: 25199783 PMC4249411

[ref45] Petersen A. M. Nielsen E. M. Litrup E. Brynskov J. Mirsepasi H. Krogfelt K. A. (2009). A phylogenetic group of *Escherichia coli* associated with active left-sided inflammatory bowel disease. BMC Microbiol. 9, 171–177. doi: 10.1186/1471-2180-9-171 19695087 PMC2736970

[ref46] Pires-dos-Santos T. Bisgaard M. Christensen H. (2013). Genetic diversity and virulence profiles of *Escherichia coli* causing salpingitis and peritonitis in broiler breeders. Vet. Microbiol. 162, 873–880. doi: 10.1016/j.vetmic.2012.11.008, PMID: 23201240

[ref47] Platell J. L. Trott D. J. Johnson J. R. Heisig P. Heisig A. Clabots C. R.. (2012). Prominence of an O75 clonal group (clonal complex 14) among non-ST131 fluoroquinolone-resistant *Escherichia coli* causing extraintestinal infections in humans and dogs in Australia. Antimicrob. Agents Chemother. 56, 3898–3904. doi: 10.1128/AAC.06120-11, PMID: 22526317 PMC3393427

[ref48] Poulsen L. L. Thøfner I. Bisgaard M. Christensen J. P. Olsen R. H. Christensen H. (2017). Longitudinal study of transmission of *Escherichia coli* from broiler breeders to broilers. Vet. Microbiol. 207, 13–18. doi: 10.1016/j.vetmic.2017.05.029, PMID: 28757012

[ref49] Rai S. Dash D. Agarwal N. (2023). Introducing the new face of CLSI M100 in 2023: an explanatory review. Indian J. Med. Microbiol. 46:100432. doi: 10.1016/j.ijmmb.2023.100432, PMID: 37945125

[ref50] Ramatla T. Ramaili T. Lekota K. E. Ndou R. Mphuti N. Bezuidenhout C.. (2023). A systematic review and meta-analysis on prevalence and antimicrobial resistance profile of *Escherichia coli* isolated from water in africa (2000–2021). Heliyon. 9:e16123. doi: 10.1016/j.heliyon.2023.e16123, PMID: 37274713 PMC10238873

[ref51] Rawla P. Barsouk A. (2019). Epidemiology of gastric cancer: global trends, risk factors and prevention. Gastroenterol Rev 14, 26–38. doi: 10.5114/pg.2018.80001, PMID: 30944675 PMC6444111

[ref52] Riley L. (2014). Pandemic lineages of extraintestinal pathogenic *Escherichia coli*. Clin. Microbiol. Infect. 20, 380–390. doi: 10.1111/1469-0691.12646 24766445

[ref53] Shetty V. P. Akshay S. D. Rai P. Deekshit V. K. (2023). Integrons as the potential targets for combating multidrug resistance in Enterobacteriaceae using CRISPR-Cas9 technique. J. Appl. Microbiol. 134:lxad137. doi: 10.1093/jambio/lxad137, PMID: 37410611

[ref54] Shin H. W. Lim J. Kim S. Kim J. Kwon G. C. Koo S. H. (2015). Characterization of trimethoprim-sulfamethoxazole resistance genes and their relatedness to class 1 integron and insertion sequence common region in gram-negative bacilli. J. Microbiol. Biotechnol. 25, 137–142. doi: 10.4014/jmb.1409.09041, PMID: 25348695

[ref55] Solgi H. Giske C. G. Badmasti F. Aghamohammad S. Havaei S. A. Sabeti S.. (2017). Emergence of carbapenem resistant *Escherichia coli* isolates producing blaNDM and blaOXA-48-like carried on IncA/C and IncL/M plasmids at two Iranian university hospitals. Infect. Genet. Evol. 55, 318–323. doi: 10.1016/j.meegid.2017.10.003, PMID: 28987805

[ref56] Stoesser N. Sheppard A. E. Pankhurst L. De Maio N. Moore C. E. Sebra R.. (2016). Evolutionary history of the global emergence of the *Escherichia coli* epidemic clone ST131. MBio 7, 02162–02115. doi: 10.1128/mBio.02162-15 PMC4807372 27006459

[ref57] Subramanian S. Rhodes J. M. Hart A. C. Tam B. Roberts C. L. Smith S. L.. (2008). Characterization of epithelial IL-8 response to inflammatory bowel disease mucosal E. Coli and its inhibition by mesalamine. Inflamm. Bowel Dis. 14, 162–175. doi: 10.1002/ibd.20296, PMID: 17941093 PMC7108638

[ref58] Swidsinski A. Khilkin M. Kerjaschki D. Schreiber S. Ortner M. Weber J.. (1998). Association between intraepithelial Escherichia coli and colorectal cancer. Gastroenterology 115, 281–286. doi: 10.1016/S0016-5085(98)70194-5, PMID: 9679033

[ref59] Tchesnokova V. L. Rechkina E. Larson L. Ferrier K. Weaver J. L. Schroeder D. W.. (2019). Rapid and extensive expansion in the United States of a new multidrug-resistant *Escherichia coli* clonal group, sequence type 1193. Clin. Infect. Dis. 68, 334–337. doi: 10.1093/cid/ciy525, PMID: 29961843 PMC6321845

[ref60] Wong M. C. Ding H. Wang J. Chan P. S. Huang J. (2019). Prevalence and risk factors of colorectal cancer in Asia. Intest Res. 17, 317–329. doi: 10.5217/ir.2019.00021, PMID: 31085968 PMC6667372

[ref61] Wu J. Lan F. Lu Y. He Q. Li B. (2017). Molecular characteristics of ST1193 clone among phylogenetic group B2 non-ST131 fluoroquinolone-resistant *Escherichia coli*. Front. Microbiol. 8:2294. doi: 10.3389/fmicb.2017.02294, PMID: 29209300 PMC5702334

[ref62] Xicohtencatl-Cortes J. Cruz-Cordova A. Cazares-Dominguez V. Escalona-Venegas G. Zavala-Vega S. Arellano-Galindo J.. (2019). Uropathogenic *Escherichia coli* strains harboring tosA gene were associated to high virulence genes and a multidrug-resistant profile. Microb. Pathog. 134:103593. doi: 10.1016/j.micpath.2019.103593, PMID: 31195111

[ref63] Xie Y.-H. Chen Y.-X. Fang J.-Y. (2020). Comprehensive review of targeted therapy for colorectal cancer. Signal Transduct. Target. Ther. 5:22. doi: 10.1038/s41392-020-0116-z, PMID: 32296018 PMC7082344

[ref64] Zeng X. Chi X. Ho B. T. Moon D. Lambert C. Hall R. J.. (2019). Comparative genome analysis of an extensively drug-resistant isolate of avian sequence type 167 *Escherichia coli* strain Sanji with novel in silico serotype O89b: H9. mSystems. 4, 00242–00218. doi: 10.1128/mSystems.00242-18 PMC6392093 30834329

[ref65] Zhao L. Zhang J. Zheng B. Wei Z. Shen P. Li S.. (2015). Molecular epidemiology and genetic diversity of fluoroquinolone-resistant *Escherichia coli* isolates from patients with community-onset infections in 30 Chinese county hospitals. J. Clin. Microbiol. 53, 766–770. doi: 10.1128/JCM.02594-14, PMID: 25520451 PMC4390631

