# Supplementary material for: Molecular epidemiology, antibiotic resistance profile and frequency of integron 1 and 2 in adherent-invasive Escherichia coli isolates of colorectal cancer patients
Source: Front Microbiol. 2024 Jun 13;15:1366719. doi: 10.3389/fmicb.2024.1366719 (PMC11208319; doi:10.3389/fmicb.2024.1366719)
Supplement: Supplementary file 1 [file Table_1.docx]

Supplementary Table S1. Characteristics of patients by study groups.

| Surveillance  n(%) | Diagnostic  n(%) | Screening  n(%) | Gender, n(%) | | | Age, mean (y) | | Groups |
| --- | --- | --- | --- | --- | --- | --- | --- | --- |
|  |  |  | female | | male |  |  |  |
| 0 | 20(66.7) | 10(33.3) | 11 | 19 | | 55.03 | CRC1(n=30) | |
| 3(2) | 17(56.66) | 10(43.34) | 16 | 16 | | 63.17 | CRC2(n=30) | |
| 27(90) | 3(10) | 0 | 12 | 18 | | 55.81 | CRChis(n=30) | |
| 0 | 10(33.3) | 20(66.7) | 13 | 17 | | 53.74 | FH(n=30) | |
| 0 | 14(46.66) | 16(53.34) | 19 | 11 | | 57.71 | Control(n=30) | |
| 30(20) | 59(39.3) | 61(40.7) | 71 | 79 | | 57.09 | Total | |
| <0.001 | | | 0.22 | | | 0.13 | p-value | |

CRC1, colorectal cancer in situ (Tis); CRC2, CRC with tumor stage T1, T2, or T3; CRChis, with CRC history; FH, with a family history of CRC

Supplementary Table S2. Antibiotic susceptibility pattern and Characteristics of the isolated adherent–invasive *Escherichia coli* (AIEC) strains.

| (%)REPL^c^ | | (%)INV^b^ | | ADH^a^ | | Antibiotics | |
| --- | --- | --- | --- | --- | --- | --- | --- |
| p | Mean ± SD | p | Mean ± SD | p | Mean ± SD |  |  |
| 0.75 | 308.1±215 | 0.99 | 0.35±0.21 | 0.84 | 1.73±0.79 | S=15 | Trimethoprim-sulfamethoxazole |
|  | 281.03±182.48 |  | 0.35±0.16 |  | 1.67±0.41 | R=9 |  |
| 0.8 | 289.92±237.78 | 0.47 | 0.38±0.21 | 0.46 | 1.5±0.56 | S=11 | Trimethoprim |
|  | 176.36 |  | 0.13 |  | 1.38 | I=1 |  |
|  | 315.44±175.46 |  | 0.34±0.18 |  | 1.88±0.76 | R=12 |  |
| 0.9 | 303.75±190.19 | 0.77 | 0.33±0.19 | 0.72 | 1.79±0.8 | S=12 | Ciprofloxacin |
|  | 327.52±370.12 |  | 0.30±0.22 |  | 1.47±0.87 | I =4 |  |
|  | 274.45±123.07 |  | 0.39±0.20 |  | 1.7±0.31 | R=8 |  |
| 0.57 | 323.46±235.67 | 0.6 | 0.32±0.23 | 0.42 | 1.5±0.64 | S=11 | Tetracycline |
|  | 171.90±47.67 |  | 0.37±0.16 |  | 1.81±0.69 | R=13 |  |
| 0.95 | 303.85±295.97 | 0.29 | 0.23±0.2 | 0.43 | 1.41±0.64 | =3 <1 | Rifampin |
|  | 297.11±193.37 |  | 0.36±0.19 |  | 1.75±0.67 | >2=21 |  |

Data were analyzed using one-way ANOVA.*, p<0.05

a Grade of adhesion. S, Susceptible; I, Intermediate; R, Resistant.

b Percentage of inoculum surviving after 1 h of gentamicin treatment (number of intracellular bacteria/initial inoculum × 100).

c Number of intracellular bacteria at 24 h post-infection/number of bacteria at 1 h post-infection × 100

Supplementary Table S3. Comparison of the presence of genes in AIEC and Non-AIEC isolates

| P | Non-AIEC:50 | AIEC=24 | Gene |
| --- | --- | --- | --- |
|  | (%)n | (%)n |  |
| 0.07 | 29(59.2%) | 19(79.2%) | int1 |
| 0.25 | 30(61.2%) | 11(45.8%) | Int2 |
|  | 19(38%) | 10(41.6%) | Int1-Int2 |

AIEC: adherent–invasive Escherichia coli.

Supplementary Table S4. MLST results of selected isolates.

| CC^a^ | ST | Allele number of Housekeeping Genes | | | | | | | Isolates |
| --- | --- | --- | --- | --- | --- | --- | --- | --- | --- |
|  |  | adk | fumC | gyrB | icd | mdh | purA | recA |  |
| 131 | 131 | 53 | 40 | 47 | 13 | 36 | 28 | 29 | 46 |
| 131 | 131 | 53 | 40 | 47 | 13 | 36 | 28 | 29 | 10 |
| 131 | 838 | 53 | 14 | 47 | 13 | 103 | 28 | 29 | 132 |
| 10 | 167 | 10 | 11 | 4 | 8 | 8 | 13 | 2 | 11 |
| 10 | 167 | 10 | 11 | 4 | 8 | 8 | 13 | 2 | 131 |
| 14 | 1193 | 14 | 14 | 10 | 200 | 17 | 7 | 10 | 72 |
| 14 | 14 | 14 | 14 | 10 | 14 | 17 | 7 | 10 | 48 |
| 73 | 73 | 36 | 24 | 9 | 13 | 17 | 11 | 25 | 101 |
| - | 135 | 13 | 39 | 50 | 13 | 16 | 37 | 25 | 40 |
| 95 | 95 | 37 | 38 | 19 | 37 | 17 | 11 | 26 | 49 |
